# Post-cold war United Nations peacekeeping operations: a review of the case for a hybrid level 2+ medical treatment facility

**DOI:** 10.1186/s40696-015-0006-z

**Published:** 2015-07-10

**Authors:** Ralph Jay Johnson

**Affiliations:** Bravo Branch, 1st BDE, 1 Southern Training Division, 75th Training Command, 10949 Aerospace Ave., Houston, TX 77034 USA

**Keywords:** Post-cold war, United Nations, Peacekeeping operations, Peacekeepers, Military medical care, Military medical treatment, Tiers, Echelons, Levels

## Abstract

Post-Cold War, UN peacekeeping operations (UN PKOs) have become larger, more mobile, multi-faceted and conducted over vast areas of remote, rugged, and harsh geography. They have been increasingly involved in dangerous areas with ill-defined boundaries, simmering internecine armed conflict, and disregard on the part of some local parties for peacekeepers’ security and role. Yet progressively there have been expectations of financial restraint and austerity. Additionally, UN PKOs have become more “robust,” that is, engaged in preemptive, assertive operations. A statistically positive and significant relationship exists between missions’ size, complexity, remoteness, and aggressive tenor and a higher probability of trauma or death, especially as a result of hostile actions or disease. Therefore, in the interest of “force protection” and optimizing operations, a key component of UN PKOs is health care and medical treatment. The expectation is that UN PKO medical support must conform to the general intent and structure of current UN PKOs to become more streamlined, portable, mobile, compartmentalized, and specialized, but also more varied and complex to address the medical aspects of these missions cost-efficiently. This article contends that establishing a hybrid level 2—a level 2 with level 3 modules and components (i.e., level 2+)—is a viable course of action when considering trends in the medical aspects of Post-Cold War UN PKOs. A level 2 medical treatment facility has the potential to provide needed forward mobile medical treatment, especially trauma care, for extended, complex, large-scale, and comprehensive UN PKOs. This is particularly the case for missions that include humanitarian outreach, preventive medicine, and psychiatry. The level 2 treatment facility is flexible enough to expand into a hybrid level 2+ with augmentation of modules based on changes in mission requirements and variation in medical aspects.

## Introduction

An essential element of United Nation’s peacekeeping operations (UN PKOs) is provision of health services in support of mission personnel in areas of operations [1]. The overall objective of medical support is the physical and mental well-being of deployed personnel, conservation of human resources, preservation of life, and limitation of residual physical and mental disabilities. A nascent understanding suggests that quality health service means that “planning, coordination, execution, monitoring, and professional supervision impacts the health and well-being of mission members and ultimately the mission’s successful execution” [2]. Arguably, peacekeepers operate better when they are healthy and know that high-quality medical treatment is available in case of injury or illness. Nevertheless, UN PKOs are becoming increasingly mobile over wide swaths of rugged/remote geographic areas to meet extensive and varied multi-dimensional mandates—all while facing considerable financial constraints [3–5]. Also, UN PKOs are operating in hostile environments with poorly defined boundaries, continued armed conflict, and scant assurance of respect for their safety.

To manage and treat sizable numbers of casualties, military medicine, including UN PKOs, uses a planning technique involving echelons (“levels”) of care to reduce the task of providing medical treatment to manageable proportions [6, 7]. Levels of care represent a framework for rational, practical, and efficient deployment of medical resources in support of missions. This framework developed from the premise that casualties occur in places remote from care facilities and these patients must be treated and sheltered at staging points before evacuation. Thus, a medical event or condition is addressed at a level of care that offers the necessary sophistication in skills and complexity in technology and materials. If it cannot be addressed at one level, the patient is moved to a higher level. Thus, a system was instituted for progressive care and five echelons of care were recognized (basic plus levels 1–4). The effectiveness of treatment is enhanced by a division of labor and standardized treatment at each echelon. Minor casualties are preempted early so that serious casualties can receive better treatment. A nagging problem with the system has been a lack of suitable facilities to treat emergencies in forward areas of operations. Casualties are transported long distances with consequent risk. The Medical Support Manual for United Nations Peacekeeping Operations (2nd ed.) notes that “levels of medical support have been standardized to ensure the highest standards of peacekeeping medical care come from different countries with varying standards … medical support plans must be designed to meet specific operations demands” [1].

In this system, the level 2 mobile medical hospital, though mobile, portable, and flexible, is the consequence of deploying a tiered system that evolved from experience in sustaining sizable pre-Cold War UN PKOs that were relatively fixed/static and linear. However, reliance on level 2 mobile facilities is also the consequence of a tiered system with increasing complexity that also can rely on the level 3 fixed-facility hospital (or transport out-of-country to such a facility) for extensive diagnostics, trauma surgery, specialist care, and long-term medical care capabilities [8, 9]. In some cases, level 3 facilities have not been deployed and their medical capabilities have rarely been deployed fully. Such care has been obtained from an existing civilian hospital in or near the area of operations or a military hospital in a neighboring stable nation [1].

Previously, UN PKO components had their own basic, level 1, and level 2 medical personnel. Nevertheless, UN PKO missions have attempted to provide a standalone, central level 2 mobile hospital from a troop-contributing nation. These facilities, while relatively fixed/static, can provide mobile care in dynamic operations [10]. However, as the complexity of operations has increased, the need to fix quarters and provide more sophisticated medical capabilities has also increased, thus decreasing mobility. It has always been difficult to balance capabilities and mobility. Smaller mobile medical facilities still need some assets and personnel assigned to larger fixed facilities and remote hospitals despite sacrificing some mobility. That is, to offset any reduction in mobility, the most mobile and mission-essential assets from level 3 augment level 2 and even level 1 [11].

That said, the level 2 mobile hospital remains the standard, mainstay, workhorse, and lynchpin for relatively large-scale UN PKOs in various theaters. The result is lower levels of medical care relying heavily on non-organic transportation personnel to ensure movement and through-put so that care arrives where needed and need is transported to where care can be provided [12, 13]. Movement, in particular patient transport, always incurs risks. Furthermore, UN PKOs are increasingly involved in remote “failed states” [14]. The local in-country hospitals that could have been used for level 3 care—and even remote military hospitals in neighboring so-called stable nations—are compromised. Their use for UN personnel is not feasible in that “they are poorly equipped and incapable of providing many basic services, medical emergent stabilization services are limited, blood supplies are unavailable, tainted, and even non-existent, and medicines, if even available, are expired, and there is poor sanitation and hygiene” [15]. This situation leads to even lengthier and more expensive transports for relatively routine medical procedures as well as catastrophic emergencies needing higher than level 2 care. This then overburdens the transport system; it also affects peacekeeper downtime when peacekeepers cannot return to duty quickly after routine procedures.

The situation is even further complicated because UN PKO personnel operate in environments marked by poor sanitation, as well as water- and food-borne vectors and illnesses, and are at high risk for infectious and debilitating diseases [16–20]. Thus, preventive medicine—a level 3 commodity—is absolutely necessary in remote areas of operation (at level 2 or 1) to advance force protection and civil-military cooperation.

The hope is that UN PKOs will become streamlined, faster, more efficient, and more capable so as to be involved in more wide-ranging, multi-faceted operations conducted over large, diverse, and remote geographic areas. Simply put, “there are hopes to structure slow and cumbersome processes to quickly respond to emergent crises” (cf. [21]). Consequently, medical support is also likely to conform to the larger structure of the UN PKO. Medical support will become smaller, more portable and mobile, more compartmentalized, and more specialized (i.e., modular) to establish itself more quickly in areas of operations. Also, in response to harsh and unforgiving environments, UN PKOs may need to become more self-sufficient and insular (cf. [22]).

Therefore, through a review of open sourced research and doctrine, this article contends that a hybrid level 2 medical treatment facility augmented with proper services and modules is the most promising option when accounting for post-Cold War trends in the medical aspects of UN PKOs. Risk and trauma have been escalating rapidly, whereas the response has come too late. It is hoped that this report will enable planners and policymakers in more quickly enhancing medical support to UN PKOs and better ensure the health and well-being of UN PKO members, which has increasingly included among the world’s militaries. Better health and well-being of UN PKO members is the ideal in medical support ensconced in the UN’s medical mission statement.

## Review

### Medical aspects

Johnson [23–25] reported on the medical aspects of post-Cold War UN PKO missions, summarized as follows.

1. UN PKOs are expanded and more robust in post-Cold War affairs. They have been and can expect to (1) be conducted in the world’s rugged, inhospitable, and inherently dangerous peripheries, primarily on the margins of the Third and Fourth Worlds, (2) involve less benign handovers from unfinished security missions, (3) have questionable consent by belligerents to the peace process and simmering conflicts ready to boil, and (4) draw peacekeepers into hostile action or becoming victims of remote explosive devices.
2. The medical events that UN PKO medical support staff encounters, therefore, will increasingly resemble combat trauma and include exotic and debilitating illnesses.
3. Thus, the conundrum that medical commands face is whether to (1) move medical treatment to patients in the forward area of operations and risk their medical personnel, (2) move patients back to medical treatment with risk to the patients, or (3) strike a practical compromise in between.
4. Additionally, the peripheries in which post-Cold War UN PKOs operate have their own locally endemic exotic diseases, other inhospitable and risky features, and wicked flora and fauna. These all pose risks to UN peacekeepers, including medical personnel. This demands preventive medical measures throughout the area of operations. Also, one thing is certain, civil conflict and its ancillary human tragedies are not good for the human psyche. Thus, psychiatric medicine is needed.
5. Furthermore, the demand for civil/military-driven humanitarian medical outreach far into the margins of already risky and dangerous situations adds a new and dangerous dimension to UN PKO missions and their medical components.
6. This creates a need for generalist/family practice medical professionals and medical sub-specialties, particularly surgical sub-specialties. As civilians—in particular, women and children—are caught in the middle of all this, there will be more emphasis on women’s and family health issues. So there is an expanded need for women health professionals and family practitioners, in particular in cultures that prohibit cross-gender contact.
7. In addition, as expanded post-Cold War UN PKO missions grind on, attrition in medical staffs will require a reserve back-fill system. The downside is that deployed augmentees are at higher risk for psychological morbidities and may be less physically fit for in-the-field austerities.

### Need for a modified level 2 medical treatment facility

The literature on medical aspects of expanded and more robust post-Cold War UN PKOs suggests the need for field medical treatment support capable of moving itself, while retaining the ability to deliver varied medical care at different levels. This must be accomplished to provide medical treatment in far forward areas of operations, especially trauma care, which sustains (1) extended and comprehensive operations, (2) maneuvers, and (3) humanitarian outreach. A modified UN PKO level 2 facility can provide the necessary bed capacity for trauma and intermediate care for casualties resulting from hostile action, injuries, and illness [1, 26]. This facility can be variable enough to expand to include augmentation by medical detachments in response to changing mission requirements. Additionally, this component’s far forward medical care should be upgraded consistent with U.S. and British models with medics who are (1) assigned to patient transport and other medical duties and (2) trained and certified as emergency medical technicians and paramedics or emergency practical nurses [9, 27]. For forward area units, designated squad-level military personnel should be trained and certified as combat life savers or in basic first aid. The objective is to enhance and push medical/trauma care knowledge and skills down to the forward areas of operations to improve responsiveness closer to medical emergencies.

Mobility is important as medical support must move in tandem with forward area of operations peacekeepers. The level 2 facility technically is mobile and provides fundamental trauma care as a base on which modules can be “plugged in for play” (i.e., modularity). Specifically, this allows other specialty components to be added or subtracted as needed to offer augmentation tailored to the mission and its changing nature [28, 29]. Thus, the next generation of medical care for UN PKOs can have the flexibility to expand (or contract) to support needed medical detachments, while retaining mobility and the ability to provide comprehensive medical care, including trauma care. Note, modularity has been used and incorporated in US Armed Forces and NATO/UE procedures and doctrine and it cannot be over-emphasized that modularity of medical support is not a new concept and has been developed by US Armed Forces and NATO/UE to meet the challenges of their missions that bear striking similarity to the challenges UN PKOs have been increasingly facing in the Post-Cold War [1, 30–34]. Rather, what is relatively innovative is advocating modularity’s adoption, adaptation to and expanded and practical use for medical support on UN PKOs to overcome new challenges regarding the health nuances of Post-Cold War UN PKOs. (Note: UN PKOs have never been restricted from using modular medical support either).

Level 2 facilities are in areas of operations where medical personnel are best able to intervene medically to alter what might otherwise be debilitating or fatal medical outcomes [28, 29]. They also can be augmented with health providers to care for common and endemic diseases and psychological stressors encountered in operations [9]. Level 1 components are primarily designed to treat life-threatening conditions and evacuate to level 2, possibly even level 3 or out of the country [1]. Note that under the changing conditions of UN PKO missions, level 1 medical facilities are insufficient to provide the level of care necessary to avoid out-of-country medical evacuation and repatriation [5, 15]. Level 2 can provide trauma care and basic medical treatment, as well as hold patients who can return to duty, and surgical teams, preventive medicine teams, and psychiatric providers alone or in combinations can be plugged into the level 2 facility depending on operations, although maintaining them at a level 3 facility is more traditional. Level 2 has no intensive care, medical supply, or extensive formulary capability [1]. However, through augmentation, level 2 can provide limited specialty care and limited pharmacy, blood, medical supply, and equipment capability. Level 2 also lacks effective operations planning, patient administration, and signal support—components that only a level 3 facility provides. It can also accommodate components to address humanitarian outreach and women’s healthcare.

An ad hoc/hybrid level 2 facility can provide care comparable to a level 3 but cannot supplant the level 3 entirely. Future operations’ mission structures, such as the post-Cold War expanded structures, will require basic and level 1 / 2 augmented support and need to be farther forward and nearer field units. Level 2 should acquire medical care aspects of level 3 such as preventive medicine and psychological care providers. A condensed level 3 can then concentrate on medical command for overall operations. Mission operations needs support from a level 3 facility to provide administration and coordination. Therefore, one level 3 facility—but a condensed level 3—should be assigned per mission at the command level.

In future post-Cold War expanded UN PKOs, the level 2 facility should be modular with the ability to expand with add-on level 3 components. This aligns with the provision of medical treatment and specialized care based on operations’ exigencies, hostilities, patients, terrain, and civil-military concerns. To support such UN PKOs, the unit must be tactical. Therefore, it must have the maneuverability to rapidly travel over some distance in tandem with peacekeepers.

The level 2 facility arguably is the center of gravity for medical support for UN PKOs. In field operations, this component of care provides seriously hurt and ill patients with life-saving medical intervention. It is also where patients are stabilized for evacuation out-of-operations and even out-of-country. Thus, ground and air Medevac capabilities with highly trained medical personnel for en-route life support must still be emphasized. The UN PKO medical literature regarding PKO medical aspects suggests that future medical support should be able to move quickly over large distances and provide comprehensive coverage farther forward in tandem with mobile forward area peacekeepers. Augmented level 2 facilities provide the level of care to do this effectively. Recent operations have demonstrated that the focus of level 2 can be trauma care, followed by non-hostile injuries and then illness (if augmented by preventive medicine teams) (cf. [9, 28, 29]).

Level 3 can continue to offer specialized surgical capabilities. It can also have the command-staff and extensive medical logistics and maintenance capabilities required for sustained long-term care. Its drawback is that it lacks modular flexibility. Therefore, given the expected direction of future UN PKOs, emphasis on medical support for operations must be at the level 2 facility that also incorporates (1) preventive and psychological medicine, (2) portability and mobility, (3) medical sustainment pushed as far forward as possible, and (4) accommodation for expanded specialized care responsive to operational contingencies (e.g., humanitarian outreach and women’s healthcare). This also permits modular expansion of bed capacity for larger treatment, holding, and evacuation.

### The mobile modular level 2+

In UN PKOs, personnel and material are available for level 2+ as attachments and augmentation building blocks for the current level 2. Level 2+ should have trauma and medical clinic (general practice) services, nursing services with psychiatric nursing resources (i.e., “dual hats”), expanded pharmacy, blood supply, and expanded medical stock. Preventive medicine teams should be attached—but dispatched and roving throughout the theater—to address field sanitation issues, potable water acquisition, disease prevention, and non-hostile action injury reduction (cf. [16]).

The command structure should retain the rank-and-staff structure of the level 2 facility with slight elevation due to its add-ons from level 3. The level 2 facility should retain the capability to expand and exceed normal bed and sub-specialty capability through modularity. It should have a commander, deputy for clinical services, director for nursing care, and administrative officer [1]. This facility should not be an amalgamation from different troop-supplying nations. Rather, it should be a unified, cohesive entity from one troop-supplying nation, capable of maintaining its own vehicles, generators, and equipment and conducting basic medical maintenance. Also, this unit must be able to communicate with field units, aviation, and ground transportation and lower and higher elements. Logically, clinical services should be organized around trauma, resuscitation, and life-and-limb and eyesight sustainment with the intent to support continuous operations. There should be a provision for general and orthopedic practice as well but not extensive diagnostic capabilities, which can be maintained at level 3. At the level 2 facility, the need for trauma care is obvious, as is the need to treat most illnesses encountered in operations. This includes emergency medical treatment. Nursing services should include bed care and patient specialists. Service and supply should allow the organization to increase in size threefold. That way, if the level 2 cannot address a particular challenge, it can be expanded modularly by adding on those capabilities. It must be underscored that Post-Cold War US and NATO/EU missions and operations clearly have shown multi-nationality and sharing medical assets and appropriate practitioners combined through modularity is included in their doctrine and has contributed to the outstanding, successful medical support on their missions [30–36].

To address personnel turnover, back-fill from individual augmentees (e.g., reservists and Home Guard) may be necessary. The troop-contributing nation should manage training and pre-deployment readiness. Detachments should be from either the troop-supplying nation of the level 2+ facility or modules representing one troop-supplying nation [13]. This ensures the cohesion that develops from training and working together over time and avoids communication difficulties related to multi-national amalgamations. These modules would be capable of relatively rapid deployment. Expansion of the level 2+ would also give level 2+ leaders opportunities to develop leadership skills, work on tactical field problems, and meet operational challenges in the field [9]. Additional non-unit equipment should be available from the level 3 to the level 2+ depending on mission needs.

**Fig. 1:**
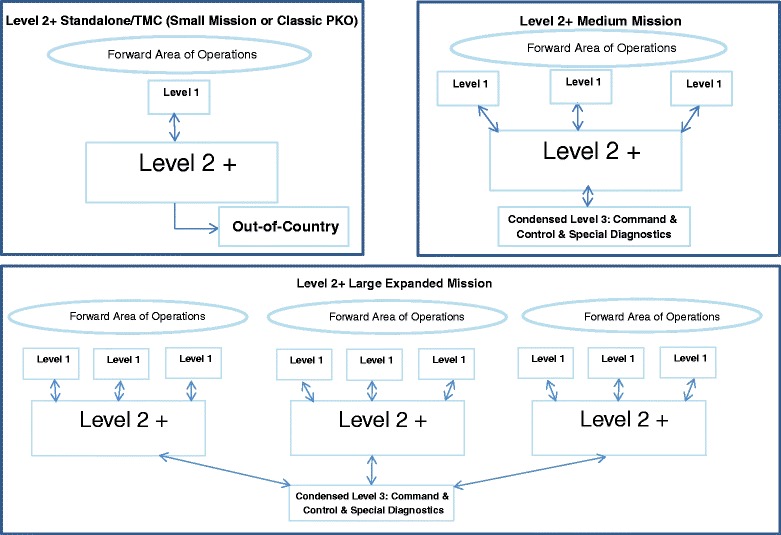
Possible Level 2 + Medical Treatment Facility PK Mission Configurations

Detachments such as humanitarian medical aid could be organized within the level 2+ and/or drawn on from the level 3. This system can facilitate a modular push forward. This would improve adaptability necessary for future UN PKOs that might have different and more particular requirements, for example: those with specific “hinterland” family medicine humanitarian outreach mobile clinics as described by Milosevic [37]; or, preventive medicine “taskings” in the peripheries as described by Hazra [16]. These modules would require fewer personnel and less equipment and be interchangeable. Ideally, several level 2 + s could be fielded in large-scale missions and coordinated by the condensed but refocused level 3 (see Fig. 1).

Supporting this shift in medical treatment facilities, and furthering rapid deployment, will require pre-positioning, depot, cache, tracking and management, maintenance of equipment, and contracting and finance specialists. As the Liberian (ECOWAS) mission reported, every contingent, including medical units and treatment facilities, must be insular and self-sufficient such that it includes laundry, catering, accommodations, office, communications, explosive ordinance disposal (EOD), and observation [38]. This means they must be equipped to support whatever type of medical care mission they encounter. This might require extra inventory or specialized inventory for plug-in-and-play, depending on operational requirements. This also would create a redundancy of material that has proved useful in previous and prolonged operations that resulted in the need for replacement and maintenance of worn and broken equipment.

## Conclusions

**Fig. 2:**
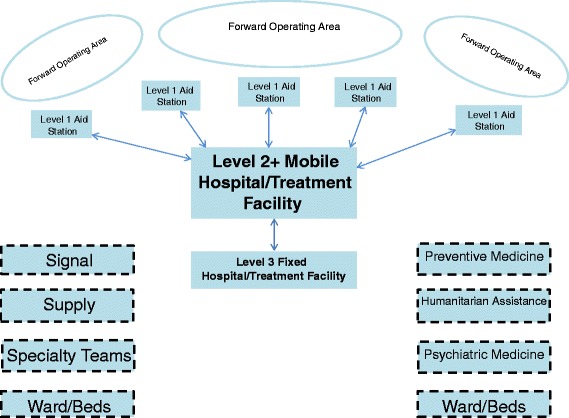
Possible Theater Level 2 + Medical Treatment Facility for PK Mission(s) with Modules for Attachment or “Plug-in-and-Play”

Predicting the future is precarious. Nevertheless, the evidence-based work reported here suggests that a hybrid level 2+ medical facility augmented with proper services and modules is optimal and best-practices for addressing trends and medical aspects of future UN PKOs (see Fig. 2).

Figure 2 basic and level 1 medical care is a critical capability required for all UN PKOs. Level 2 is where wounded, injured, or ill patients are collected, stabilized, and treated prior to return to duty or evacuation. If past experience predicts future experience, future UN PKOs’ medical support will be mobile across large distances into dangerous, remote, and rugged areas. Medical care will be close-in to facilitate rapid treatment and evacuation from basic and level 1 facilities. In previous operations, it was unrealistic to rely on aero-evacuation entirely, in particular with (1) constant disruption due to inclement weather, (2) a lack of landing areas and night vision devices, and (3) even hostile anti-aircraft fire. The requirement will be a more complex and larger level 2 facility capable of moving closer to field operations. A consideration for planners will be security for medical treatment facilities in areas where conflict resolution is indeed robust. This is because the level 2 facility is a potentially vulnerable, high-value target. Another consideration is that additional modules will reduce mobility even with more transportation components. Thus, the level 2 must have more of its own transportation with the inclusion of additional modules.

The recommendations discussed here have the potential to provide medical care capable of supporting UN PKOs with more mobile medical capabilities, but without the footprint of a level 3 or the need for expensive out-of-country facilities. The facility will have an appropriate size to support ad hoc small contingency operations and humanitarian outreach or special operations. The level 2+ should be an element of a troop-lending nation, train as a unit, and deploy and function intact to avoid language barriers and communication issues. [12, 13]. To interface with other personnel/units on the mission who speak a different language, interpreters may be needed. These smaller hospitals will be more easily deployed and enhanced through modularity. Modularity is achievable by implementing a system of detachments for requirements and specialties. These can come from the level 2+’s troop-lending nation or self-contained detachment modules from other troop-lending nations. The point is that standards of care must be comparable and personnel must train and work together in the modules. More importantly, this provides the mission commander with flexibility in planning for particular and varied missions or in responding to mission changes.

The lessons reported here convey the importance of medical care to UN PKOs. They suggest courses of action that include use of modular augmentation in the level 2 medical care for UN PKOs. This system could significantly enhance the provision of military medical care on UN PKO missions in ways that are capabilities-based and efficiently achieved with flexibility, mobility, adaptability, and expediency to accommodate particular, varied, and evolving UN PKOs. Given the direction of post-Cold War UN PKO trends, the recommendations in this report should be incorporated across the spectrum of medical aspects of expanded, post-Cold War-type missions. The operational component of the level 2 hospital can be modularly augmented with detachments and teams, which can be plugged in for play. At its core, it will still provide needed trauma and general practice care. The importance of the work reported here is that it provides a general conceptual approach to considerations of medical aspects across the spectrum of UN PKO missions, specifically the iterative identification of relevant medical and health aspects to UN PKO missions and planning for medical controls accordingly.

In light of the changed in the nature of Post-Cold War PKOs, the argument for a change in terms of policy revision and adoption for PKO medical support described herein is a natural logical extension. The next logical extension in terms of ‘what is to be done’ are steps to marshal this along. The following are possible generic steps, in a general sequential order but conducted iteratively, that at minimum can be aimed to mobilize policy and opinion.

- Study the requirements/standards that need to be in policy.
- Conduct and take into account a needs/risk assessment.
- Align the policy with previous policy and discard or revise un-useful policy with new policy.
- Involve others, especially stakeholders, in drafting of policy for maximum buy-in and to catch things in error or unworkable.
- Get the policy approval from the high-ranking stakeholders.
- Train and manage expectations on the necessity and benefit of the policy.
- Take care to modify and update continuously as the policy is piloted, vetted and implemented [2, 11, 39–45].

Also, there must be an assessment to the extent that recommendations grab the attention of policy makers.

One lesson learned is that UN PKO medical support planning is complex; it requires more than a simple fix of additional mobility or closer proximity or the addition of this or that facet or module. Therefore, medical support plans must be purpose built for each operation. Plans (and planners) must be capable of speedy initial reaction and sufficiently flexible to manage rapidly changing demands (cf. [2]). Thus, given the experience of past UN PKOs, in addition to fundamental structural modifications, future UN PKOs will need to adopt a medical support planning mind-set that is informed by continuously updated research and aims to achieve best practices (cf. [12, 13]). The mind-set adopted for medical planning must consider and seek to strike a delicate balance in the trinity of proximity to medical care, time involved in transporting, and security given the nature of UN PKOs. Medical capabilities must correspond to the mission, strength, and composition of the force they support and the assessed environmental hazards they face.

Finally, the paucity of research on which to base an evidence-based review of structural and procedural modifications for UN PKOs and non-war military interventions literally cries for the need for more open-sourced, peer-reviewed work in this arena. Not only does the peer-review process add credibility to findings and patina to the finished product, but incorporation into and citations in other research and critical response are indicators of the extent to which the recommendations actually have engaged policy makers.
